# The genome sequence of the bishop’s mitre shieldbug, *Aelia acuminata *(Linnaeus, 1758)

**DOI:** 10.12688/wellcomeopenres.17400.1

**Published:** 2021-11-24

**Authors:** Liam Crowley, Maxwell V.L. Barclay, University of Oxford and Wytham Woods Genome Acquisition Lab, Natural History Museum Genome Acquisition Lab, Darwin Tree of Life Barcoding collective, Wellcome Sanger Institute Tree of Life programme, Wellcome Sanger Institute Scientific Operations: DNA Pipelines collective, Tree of Life Core Informatics collective, Darwin Tree of Life Consortium

**Affiliations:** 1 Department of Zoology, University of Oxford, Oxford, UK; 2 Department of Life Sciences, Natural History Museum, London, UK

**Keywords:** Aelia acuminata, bishop’s mitre shieldbug, genome sequence, chromosomal, Hemiptera

## Abstract

We present a genome assembly from an individual male *Aelia acuminata* (the bishop’s mitre shieldbug; Arthropoda; Insecta; Hemiptera; Pentatomidae). The genome sequence is 1,170 megabases in span. The majority of the assembly (99.78%) is scaffolded into 8 chromosomal pseudomolecules, with the X and Y sex chromosome assembled.

## Species taxonomy

Eukaryota; Metazoa; Ecdysozoa; Arthropoda; Hexapoda; Insecta; Pterygota; Neoptera; Paraneoptera; Hemiptera; Heteroptera; Panheteroptera; Pentatomomorpha; Pentatomoidea; Pentatomidae; Pentatominae; Aelia; *Aelia acuminata* (Linnaeus, 1758) (NCBI:txid1511221).

## Background

The Bishop's mitre shieldbug, *Aelia acuminata*, is a common mid-sized (8-9 mm) shieldbug. The vernacular name derives from the characteristically pointed head and pronotum forming a shape reminiscent of its namesake. It is common in grassland habitats across Europe, North Africa, the Middle East and Northern Asia. In the UK it is common and widespread across the south, and can be found in most dry grassland habitats. It feeds on the seeds of a range of grasses in the Poaceae family and may be a minor pest in cereal fields ( Vaccino *et al*., 2017). It is univoltine, with adults mating and laying eggs in the spring and early summer. Nymphs develop through five instars throughout the summer months and reach adulthood by August. *Aelia acuminata* overwinters as an imago, with the increased photoperiod a key factor in termination of diapause in the spring ( Hodek, 1971).

## Genome sequence report

**Figure 1.**
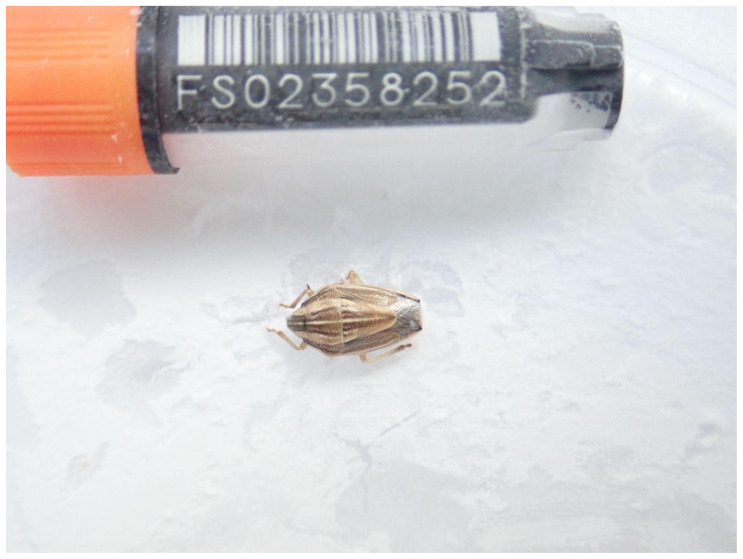
Image of the *Aelia acuminata* specimen (ihAelAcum1) used for genome sequencing, taken during preservation and processing. The specimen is shown alongside a FluidX sample tube 43.9 mm in length.

The genome was sequenced from one male *A. acuminata* ( Figure 1) collected from Wytham Woods, Oxfordshire (biological vice-county: Berkshire), UK (latitude 51.771, longitude -1.338). A total of 31-fold coverage in Pacific Biosciences single-molecule long reads and 24-fold coverage in 10X Genomics read clouds were generated. Primary assembly contigs were scaffolded with chromosome conformation Hi-C data. Manual assembly curation corrected 241 missing/misjoins and removed 25 haplotypic duplications, reducing the assembly size by 0.73% and the scaffold number by 67.21% and increasing the scaffold N50 by 132.84%.

**Figure 2.**
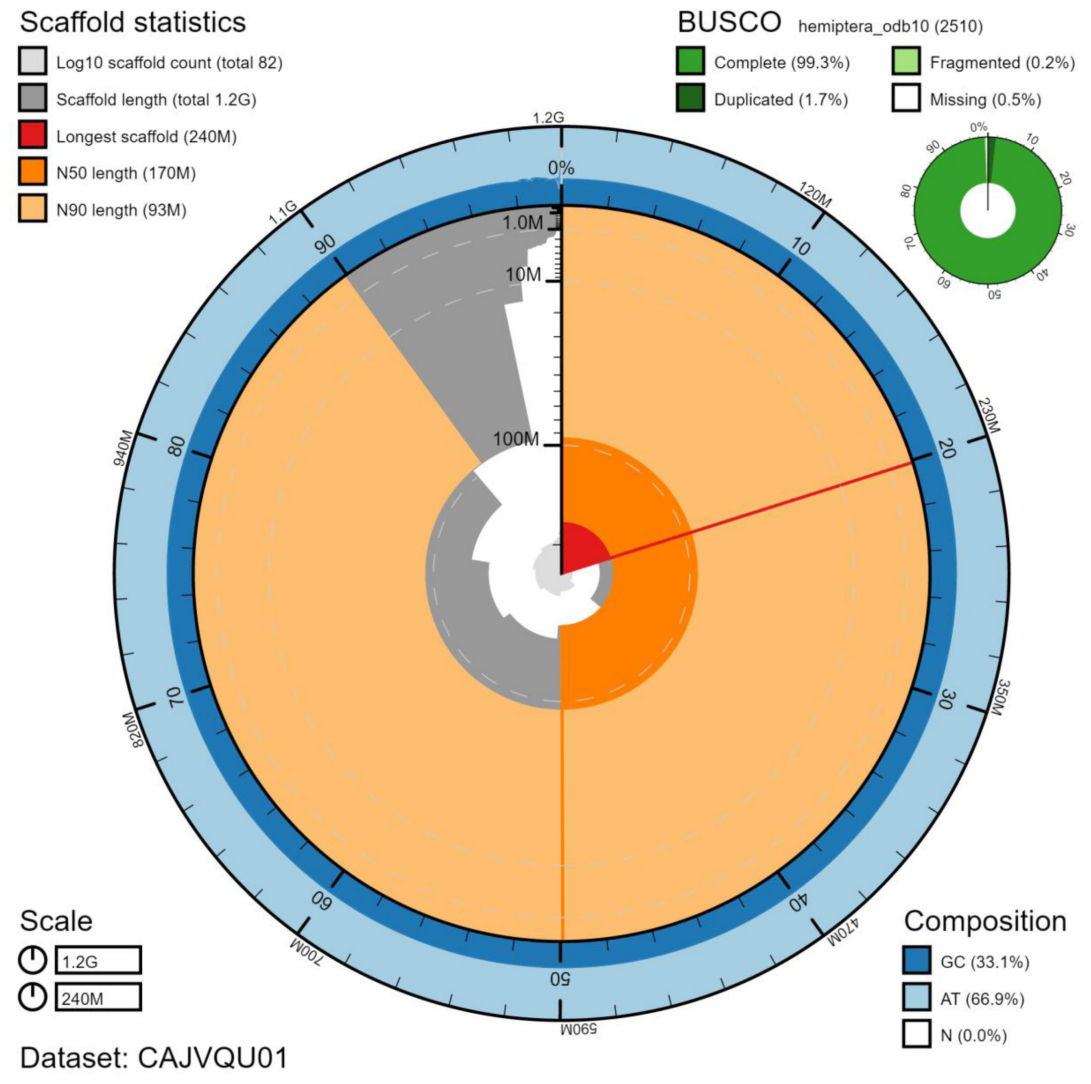
Genome assembly of *Aelia acuminata*, ihAelAcum1.1: metrics. The BlobToolKit Snailplot shows N50 metrics and BUSCO gene completeness. The main plot is divided into 1,000 size-ordered bins around the circumference with each bin representing 0.1% of the 1,170,046,398 bp assembly. The distribution of scaffold lengths is shown in dark grey with the plot radius scaled to the longest scaffold present in the assembly (235,202,771 bp, shown in red). Orange and pale-orange arcs show the N50 and N90 scaffold lengths (172,186,306 and 93,278,539 bp), respectively. The pale grey spiral shows the cumulative scaffold count on a log scale with white scale lines showing successive orders of magnitude. The blue and pale-blue area around the outside of the plot shows the distribution of GC, AT and N percentages in the same bins as the inner plot. A summary of complete, fragmented, duplicated and missing BUSCO genes in the hemiptera_odb10 set is shown in the top right. An interactive version of this figure is available at https://blobtoolkit.genomehubs.org/view/ihAelAcum1.1/dataset/CAJVQU01/snail.

The final assembly has a total length of 1,170 Mb in 81 sequence scaffolds with a scaffold N50 of 172 Mb ( Table 1). The majority of the assembly sequence (99.78%) was assigned to 8 chromosomal-level scaffolds, representing 6 autosomes (numbered by sequence length), and the X and Y sex chromosome ( Figure 2– Figure 5; Table 2). The assembly has a BUSCO v5.1.2 ( Manni *et al*., 2021) completeness of 99.3% (single 97.6%, duplicated 1.7%) using the hemiptera_odb10 reference set. While not fully phased, the assembly deposited is of one haplotype. Contigs corresponding to the second haplotype have also been deposited.

**Table 1.** Genome data for *Aelia acuminata*, ihAelAcum1.1.

*Project accession data*	
Assembly identifier	ihAelAcum1.1
Species	*Aelia acuminata*
Specimen	ihAelAcum1 (genome assembly, Hi-C); ihAelAcum5 (Hi-C)
NCBI taxonomy ID	NCBI:txid1511221
BioProject	PRJEB45203
BioSample ID	SAMEA7520372
Isolate information	Male, whole organism (ihAelAcum1); unknown sex, head/thorax (ihAelAcum5);
*Raw data accessions*	
PacificBiosciences SEQUEL II	ERR6406217, ERR6544656
10X Genomics Illumina	ERR6054995-ERR6054998
Hi-C Illumina	ERR6054999, ERR6055000
*Genome assembly*	
Assembly accession	GCA_911387785.1
Accession of alternate haplotype	GCA_911387705.1
Span (Mb)	1,170
Number of contigs	691
Contig N50 length (Mb)	4.7
Number of scaffolds	81
Scaffold N50 length (Mb)	172.2
Longest scaffold (Mb)	235.2
BUSCO * genome score	C:99.3%[S:97.6%,D:1.7%],F:0. 2%,M:0.5%,n:2510

*BUSCO scores based on the hemiptera_odb10 BUSCO set using v5.1.2. C= complete [S= single copy, D=duplicated], F=fragmented, M=missing, n=number of orthologues in comparison. A full set of BUSCO scores is available at https://blobtoolkit.genomehubs.org/view/ihAelAcum1.1/dataset/CAJVQU01/busco.

**Figure 3.**
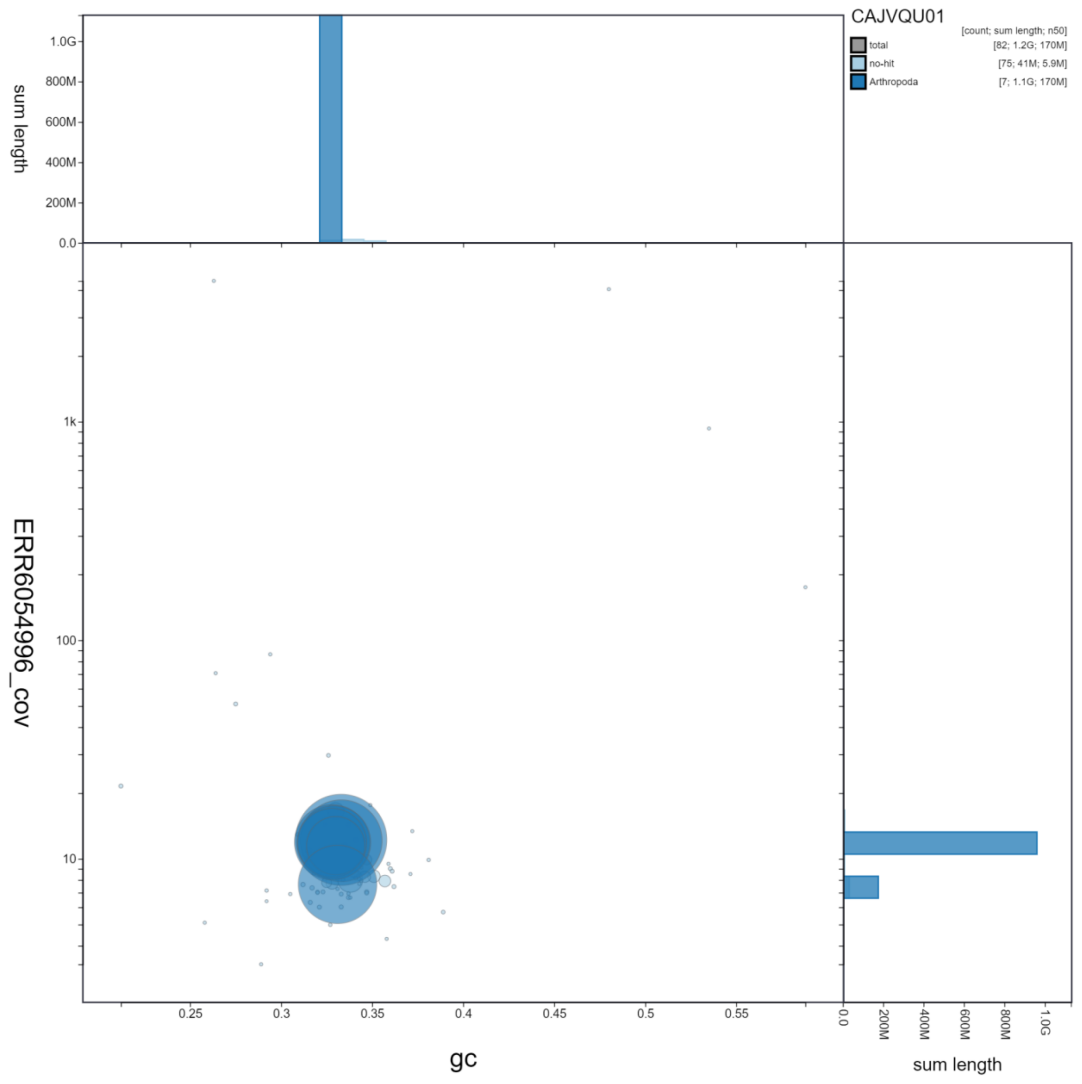
Genome assembly of *Aelia acuminata*, ihAelAcum1.1: GC coverage. BlobToolKit GC-coverage plot. Scaffolds are coloured by phylum. Circles are sized in proportion to scaffold length. Histograms show the distribution of scaffold length sum along each axis. An interactive version of this figure is available at https://blobtoolkit.genomehubs.org/view/ihAelAcum1.1/dataset/CAJVQU01/blob.

**Figure 4.**
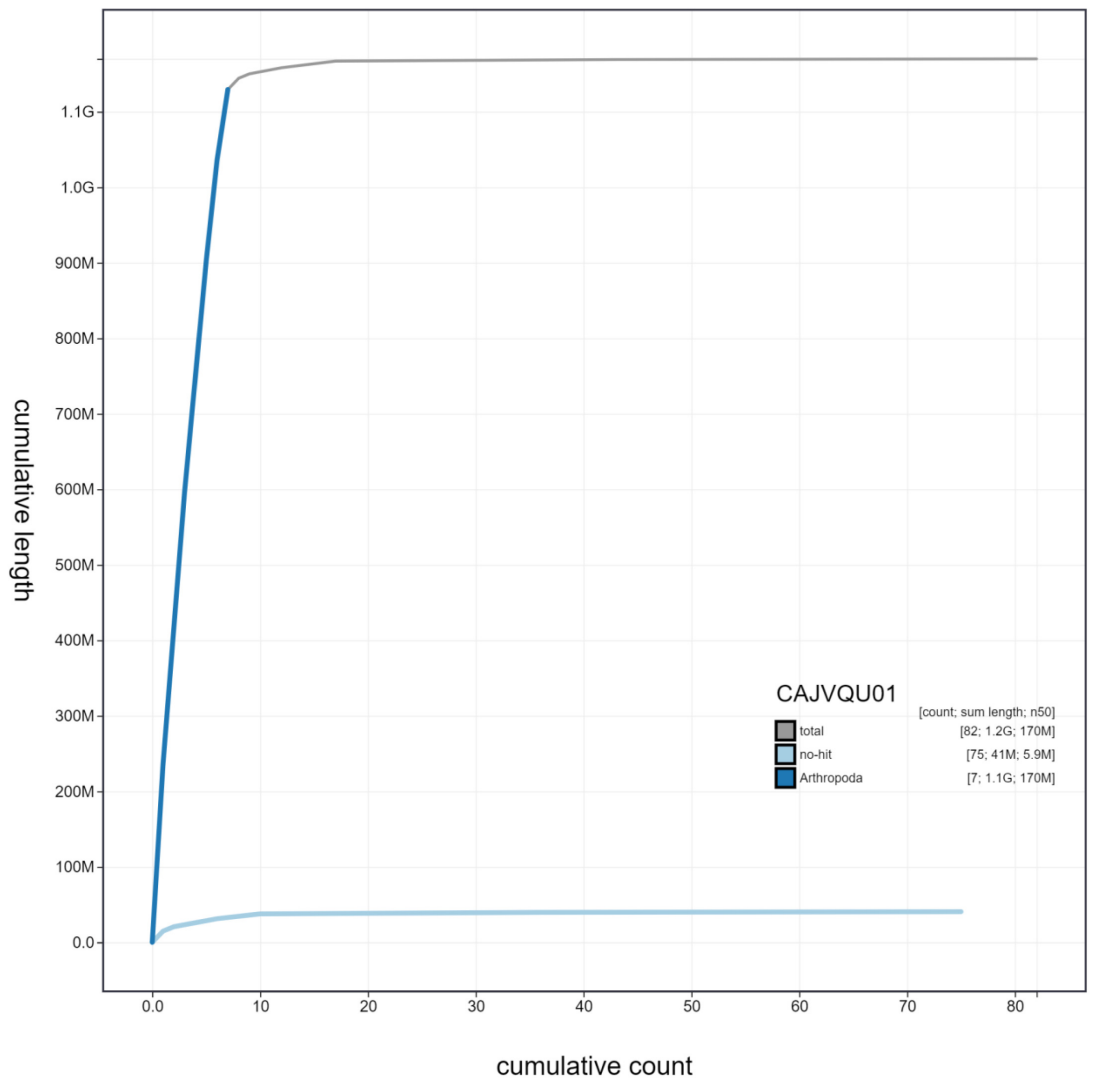
Genome assembly of *Aelia acuminata*, ihAelAcum1.1: cumulative sequence. BlobToolKit cumulative sequence plot. The grey line shows cumulative length for all scaffolds. Coloured lines show cumulative lengths of scaffolds assigned to each phylum using the buscogenes taxrule. An interactive version of this figure is available at https://blobtoolkit.genomehubs.org/view/ihAelAcum1.1/dataset/CAJVQU01/cumulative.

**Figure 5.**
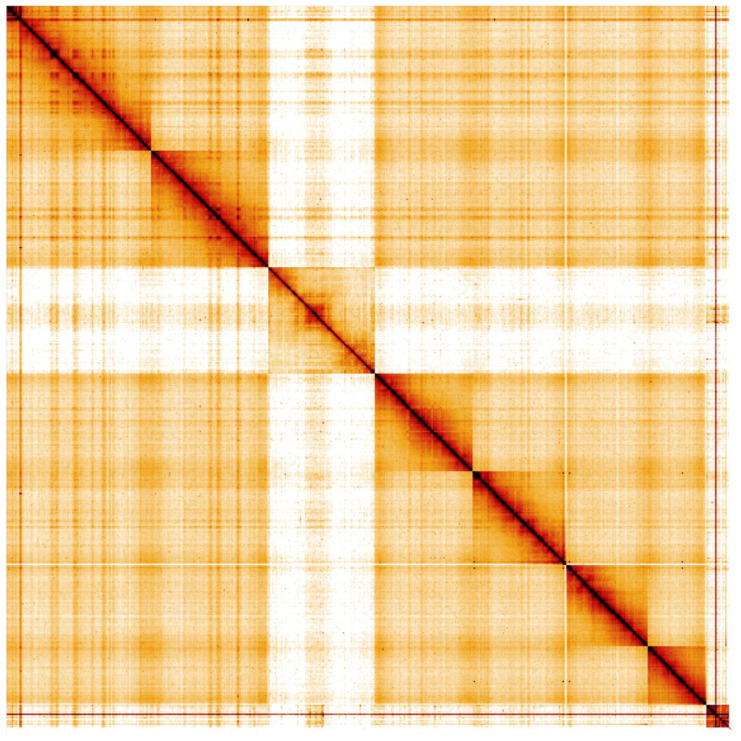
Genome assembly of *Aelia acuminata*, ihAelAcum1.1: Hi-C contact map. Hi-C contact map of the ihAelAcum1.1 assembly, visualised in HiGlass.

**Table 2.** Chromosomal pseudomolecules in the genome assembly of *Aelia acuminata*, ihAelAcum1.1.

INSDC accession	Chromosome	Size (Mb)	GC%
OU426978.1	1	235.20	33.3
OU426979.1	2	187.88	33.3
OU426981.1	3	158.11	32.8
OU426982.1	4	150.24	32.9
OU426983.1	5	132.53	32.8
OU426984.1	6	93.28	33
OU426980.1	X	172.19	33.1
OU426985.1	Y	14.82	33.9
OU426986.1	MT	0.02	26.3
-	Unplaced	25.78	33.7

## Methods

### Sample acquisition, DNA extraction and sequencing

A single male *A. acuminata* (ihAelAcum1) was collected from Wytham Woods, Oxfordshire (biological vice-county: Berkshire), UK (latitude 51.771, longitude -1.338) by Liam Crowley, University of Oxford, using a sweep net. The sample was identified by the same individual, and preserved on dry ice. A second specimen of unknown sex (ihAelAcum5) was collected from Hever Castle, Hever, Kent, UK (latitude 51.188367, longitude 0.119781) by Maxwell Barclay, Natural History Museum, by hand. The sample was identified by the same individual and was snap-frozen in liquid nitrogen.

DNA was extracted from the whole organism of ihAelAcum1 at the Wellcome Sanger Institute Scientific Operations core from the whole organism using the Qiagen MagAttract HMW DNA kit, according to the manufacturer’s instructions. Pacific Biosciences HiFi circular consensus and 10X Genomics read cloud DNA sequencing libraries were constructed according to the manufacturers’ instructions. Sequencing was performed by the Scientific Operations core at the Wellcome Sanger Institute on Pacific Biosciences SEQUEL II and Illumina HiSeq X instruments. Hi-C data were generated from the head/thorax tissue of ihAelAcum5 and whole organism tissue of ihAelAcum1 using the Arima Hi-C+ kit and sequenced on an Illumina NovaSeq 6000 instrument.

### Genome assembly

Assembly was carried out with Hifiasm ( Cheng *et al*., 2021); haplotypic duplication was identified and removed with purge_dups ( Guan *et al*., 2020). One round of polishing was performed by aligning 10X Genomics read data to the assembly with longranger align, calling variants with freebayes ( Garrison & Marth, 2012). The assembly was then scaffolded with Hi-C data ( Rao *et al*., 2014) using SALSA2 ( Ghurye *et al*., 2019). The assembly was checked for contamination and corrected using the gEVAL system ( Chow *et al*., 2016) as described previously ( Howe *et al*., 2021). Manual curation ( Howe *et al*., 2021) was performed using gEVAL, HiGlass ( Kerpedjiev *et al*., 2018) and Pretext. The mitochondrial genome was assembled using MitoHiFi ( Uliano-Silva *et al*., 2021). The genome was analysed and BUSCO scores generated within the BlobToolKit environment ( Challis *et al*., 2020). Table 3 contains a list of all software tool versions used, where appropriate.

**Table 3.** Software tools used.

Software tool	Version	Source
Hifiasm	0.15.1-r329	Cheng *et al*., 2021
purge_dups	1.2.3	Guan *et al*., 2020
SALSA2	2.2	Ghurye *et al*., 2019
longranger align	2.2.2	https://support.10xgenomics.com/genome-exome/software/pipelines/latest/advanced/other-pipelines
freebayes	1.3.1-17-gaa2ace8	Garrison & Marth, 2012
MitoHiFi	2.0 singularity	Uliano-Silva *et al*., 2021
gEVAL	N/A	Chow *et al*., 2016
HiGlass	1.11.6	Kerpedjiev *et al*., 2018
PretextView	0.2.x	https://github.com/wtsi-hpag/PretextView
BlobToolKit	2.6.2	Challis *et al*., 2020

### Ethics/compliance issues

The materials that have contributed to this genome note have been supplied by a Darwin Tree of Life Partner. The submission of materials by a Darwin Tree of Life Partner is subject to the Darwin Tree of Life Project Sampling Code of Practice. By agreeing with and signing up to the Sampling Code of Practice, the Darwin Tree of Life Partner agrees they will meet the legal and ethical requirements and standards set out within this document in respect of all samples acquired for, and supplied to, the Darwin Tree of Life Project. Each transfer of samples is further undertaken according to a Research Collaboration Agreement or Material Transfer Agreement entered into by the Darwin Tree of Life Partner, Genome Research Limited (operating as the Wellcome Sanger Institute), and in some circumstances other Darwin Tree of Life collaborators.

## Data availability

European Nucleotide Archive: Aelia acuminata (Bishop's mitre shieldbug). Accession number PRJEB45203; https://identifiers.org/ena.embl/PRJEB45203.

The genome sequence is released openly for reuse. The *A. acuminata* genome sequencing initiative is part of the Darwin Tree of Life (DToL) project. All raw sequence data and the assembly have been deposited in INSDC databases. The genome will be annotated and presented through the Ensembl pipeline at the European Bioinformatics Institute. Raw data and assembly accession identifiers are reported in Table 1.

## Author information

Members of the University of Oxford and Wytham Woods Genome Acquisition Lab are listed here: https://doi.org/10.5281/zenodo.4789929.

Members of the Natural History Museum Genome Acquisition Lab are listed here: https://doi.org/10.5281/zenodo.4790043.

Members of the Darwin Tree of Life Barcoding collective are listed here: https://doi.org/10.5281/zenodo.4893704.

Members of the Wellcome Sanger Institute Tree of Life programme collective are listed here: https://doi.org/10.5281/zenodo.5377053.

Members of Wellcome Sanger Institute Scientific Operations: DNA Pipelines collective are listed here: https://doi.org/10.5281/zenodo.4790456.

Members of the Tree of Life Core Informatics collective are listed here: https://doi.org10.5281/zenodo.5013542.

Members of the Darwin Tree of Life Consortium are listed here: https://doi.org/10.5281/zenodo.4783559.
