# Supplementary material for: The genome sequence of the bishop’s mitre shieldbug, Aelia acuminata (Linnaeus, 1758)
Source: Wellcome Open Res. 2021 Nov 24;6:320. [Version 1] doi: 10.12688/wellcomeopenres.17400.1 (PMC8817068; doi:10.12688/wellcomeopenres.17400.1)
Supplement: Peer review history

# Peer review: Reviewer response for version 1

**DOI:** 10.21956/wellcomeopenres.19237.r48195

**Published:** 2022-02-08

**Authors:** Omaththage P. Perera

**Affiliations:** 1 Southern Insect Management Research Unit, USDA, Agricultural Research Service, Stoneville, MS, USA

Genome assembly of Bishop’s Mitre Shieldbug, *Aelia acuminata *by Crowley *et al.*

 This is one of the best chromosome-scale genome assemblies available to date for a pentatomid stink bug species. Authors present the workflow clearly and I believe this genome will be an excellent reference for other pentatomid genome assemblies. I encourage authors to re-examine chromosome assignments by comparing the genome sequence to other published hemipteran genomes rather than sorting by chromosome size. This will help comparative studies down the road as more hemipteran/pentatomid genomes are sequenced and published. In addition, publishing the parameters used for various operations as a supplementary data file would be helpful to the scientific community.

Are sufficient details of methods and materials provided to allow replication by others?

Yes

Is the rationale for creating the dataset(s) clearly described?

Yes

Are the datasets clearly presented in a useable and accessible format?

Yes

Are the protocols appropriate and is the work technically sound?

Yes

Reviewer Expertise:

Genetics

I confirm that I have read this submission and believe that I have an appropriate level of expertise to confirm that it is of an acceptable scientific standard.

# Peer review: Reviewer response for version 1

**DOI:** 10.21956/wellcomeopenres.19237.r48194

**Published:** 2022-02-04

**Authors:** Surendra Neupane

**Affiliations:** 1 School of Forest, Fisheries, and Geomatics Sciences, University of Florida, Gainesville, FL, USA

The authors have presented the genome report of the *Aelia acuminata* sized 1,170 megabases with 8 chromosomal pseudomolecules using long read genome assembly. The methods for sample collection and the data analyses are technically and scientifically sound as they have utilized recent tools. The genome of *A. acuminata *can provide good addition to the Hemiptera order and baseline for the phylogenetic studies. 

**Major**: I believe CAJVQU010000004.1, and CAJVQU010000005.1 are the X and Y chromosomes. Authors have not pointed out which is X and Y chromosomes. Please provide the reason for using only One round of polishing. Other missing part in the methodology is the annotation of the genome. I would like authors to add an annotation report in the main article with information on genes and pseudogenes information. 

****

**Minor:** I was wondering if they can show the BUSCO plot in traditional figure and add ANI identity to the close related species' genomes and I would recommend authors to show them in the phylogenetic tree using single copy genes. Further, the codes used in the analysis should be deposited in the dyrad or figshare.

Are sufficient details of methods and materials provided to allow replication by others?

Yes

Is the rationale for creating the dataset(s) clearly described?

Yes

Are the datasets clearly presented in a useable and accessible format?

Yes

Are the protocols appropriate and is the work technically sound?

Yes

Reviewer Expertise:

Bioinformatics, living organism Genomics

I confirm that I have read this submission and believe that I have an appropriate level of expertise to confirm that it is of an acceptable scientific standard.

# Peer review: Reviewer response for version 1

**DOI:** 10.21956/wellcomeopenres.19237.r47419

**Published:** 2021-12-16

**Authors:** Kamil Jaron

**Affiliations:** 1 Institute of Evolutionary Biology, Ashworth Laboratories, School of Biological Sciences, University of Edinburgh, Edinburgh, UK

Crowley, Barclay *et al.,* report a genome of a hemipteran - bishop’s mitre shieldbug. The assembly is on a chromosomal scale, including identified sex chromosomes.

 The methods were described clearly, with an exception of the assignment of X and Y chromosomes, which seems to be left out of the methods section. I suppose they showed a clear signature of monoploid coverage, however, I was less sure how authors decided on which of the two monoploid chromosomes was X and which Y.

 The final assembly spans 1,170 Mb in 81 scaffolds. 99.78% of nucleotides were assigned to chromosomes, but I wondered how many scaffolds were anchored to chromosomes? Does the coverage not anchored scaffolds indicate linkage to one of the two sex chromosomes, automates or is it impossible to tell?

Are sufficient details of methods and materials provided to allow replication by others?

Partly

Is the rationale for creating the dataset(s) clearly described?

Yes

Are the datasets clearly presented in a useable and accessible format?

Yes

Are the protocols appropriate and is the work technically sound?

Yes

Reviewer Expertise:

genome evolution, non-mendelian inheritance, bioinformatics

I confirm that I have read this submission and believe that I have an appropriate level of expertise to confirm that it is of an acceptable scientific standard.
